# Supplementary material for: Pterostilbene Increases LDL Metabolism in HL-1 Cardiomyocytes by Modulating the PCSK9/HNF1α/SREBP2/LDLR Signaling Cascade, Upregulating Epigenetic hsa-miR-335 and hsa-miR-6825, and LDL Receptor Expression
Source: Antioxidants (Basel). 2021 Aug 12;10(8):1280. doi: 10.3390/antiox10081280 (PMC8389247; doi:10.3390/antiox10081280)
Supplement: Supplementary file 1 [file antioxidants-10-01280-s001.zip › antioxidants-1295469-supplementary.pdf]

Supplementary Information

Pterostilbene increases LDL metabolism in HL-1 cardiomyocytes by modulating the PCSK9/HNF1α/SREBP2/LDLR signaling cascade, upregulating epigenetic hsa-miR-335 and hsa-miR-6825, and LDL receptor expression

Disclosed by Yen-Kuang Lin 1, 2†, Chi-Tai Yeh 3,4, Kuang-Tai Kuo 5,6,†, Vijesh Kumar Yadav 3,4, Iat-Hang Fong 3,4, Nicholas G. Kounis 7, Patrick Hu 8,9 and Ming-Yow Hung 10,11,12,*

1 Biostatistics Research Center, Taipei Medical University, Taipei 110, Taiwan; robbinlin@tmu.edu.tw

2 Graduate Institute of Athletics and Coaching Science, National Taiwan Sport University, Taoyuan City 33301, Taiwan

3 Department of Medical Research and Education, Taipei Medical University-Shuang Ho Hospital,

4 Department of Medical Laboratory Science and Biotechnology, Yuanpei University of Medical Technology, Hsinchu 300, Taiwan

5 Department of Surgery, Division of Thoracic Surgery, Shuang Ho Hospital, Taipei Medical University,
New Taipei City 23561, Taiwan; doc2738h@gmail.com

6 Department of Surgery, Division of Thoracic Surgery, School of Medicine, College of Medicine,
Taipei Medical University, Taipei 110, Taiwan

7 Department of Internal Medicine, Division of Cardiology, University of Patras Medical School, 26221 Patras, Greece; ngkounis@otenet.gr

8 Department of Cardiology, University of California, Riverside, CA 92521, USA; dr.hu.md@gmail.com

9 Department of Cardiology, Riverside Medical Clinic, Riverside, CA 92506, USA

10 Department of Internal Medicine, Division of Cardiology, School of Medicine, College of Medicine,
Taipei Medical University, Taipei 110, Taiwan

11 Taipei Heart Institute, Taipei Medical University, Taipei 110, Taiwan

12 Department of Internal Medicine, Division of Cardiology, Shuang Ho Hospital,

Taipei Medical University, New Taipei City 23561, Taiwan

* Correspondence: myhung6@ms77.hinet.net; Tel.: +88-62-2249-0088; Fax: +88-62-8262-2010

† Dr. Yen-Kuang Lin and Kuang-Tai Kuo contributed equally.

* **Corresponding authors:** Ming-Yow Hung, MD, PhD, FAHA. Division of Cardiology, Department of Internal Medicine, Shuang Ho Hospital, Taipei Medical University, No.291, Zhongzheng Rd., Zhonghe District, New Taipei City, 23561, Taiwan. Tel.: +886 2 22490088; Fax: +886 2 82622010; E-mail: myhung6@ms77.hinet.net

Supplementary Table S1. Western blot antibodies sheet.

No.	Target	Dilution	Source	
1	PCSK9	1:1000	ab181142	Abcam
2	HNF1α	1:1000	ab96777	Abcam
3	HIF1α	1:1000	#36169	Cell Signaling
4	NRF2	1:1000	#12721	Cell Signaling
5	HDAC2	1:1000	#2540	Cell Signaling
6	Akt	1:1000	#9272	Cell Signaling
7	p-Akt	1:1000	#4060	Cell Signaling
8	LDLR	1:1000	66414-1-Ig	proteintech
9	SREBF2	1:1000	14508-1-AP	proteintech
10	β-actin	1:10000	60008-1-Ig	proteintech

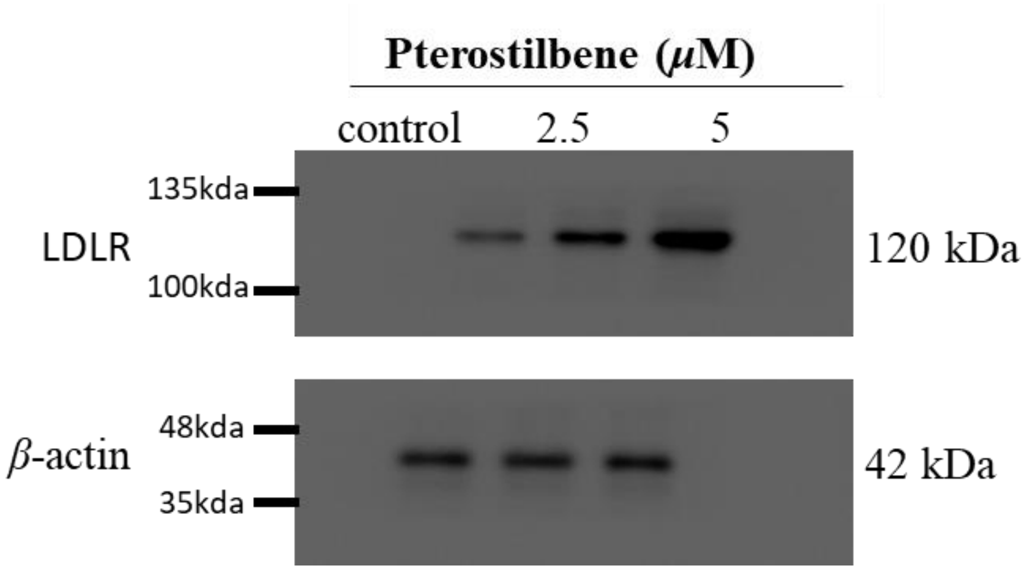

Supplementary Figure S1. Full-size blots of Figure 1B

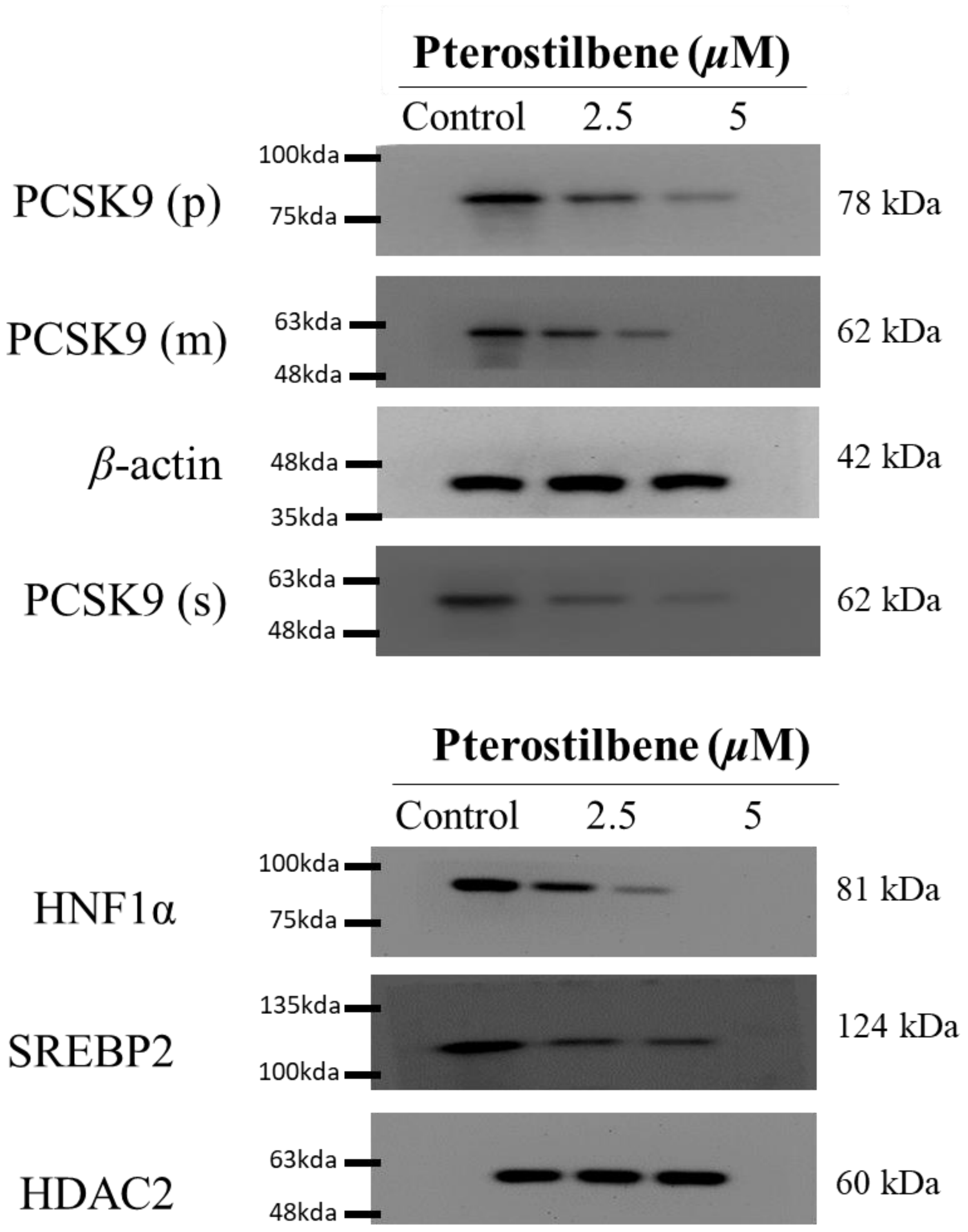

Supplementary Figure S2. Full-size blots of Figure 3A and 3D

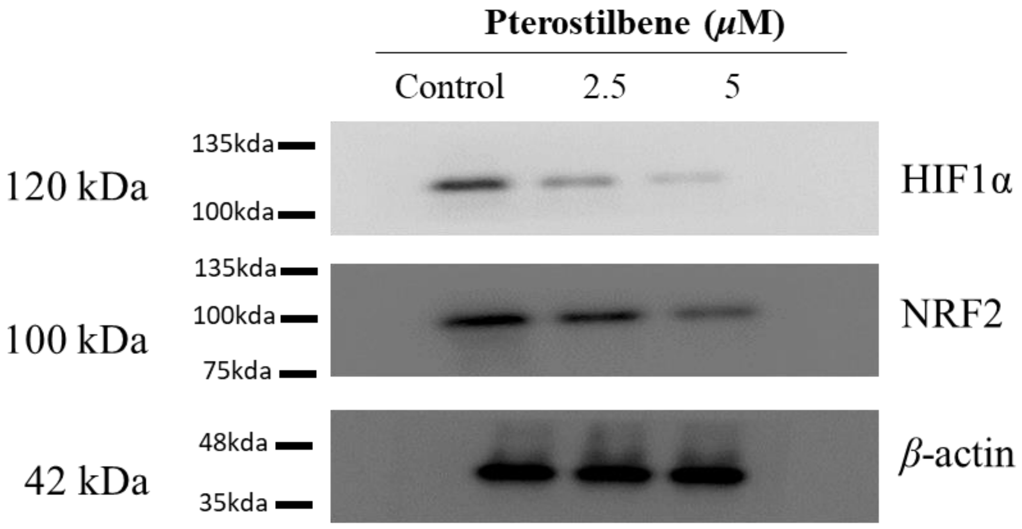

Supplementary Figure S3. Full-size blots of Figure 4D

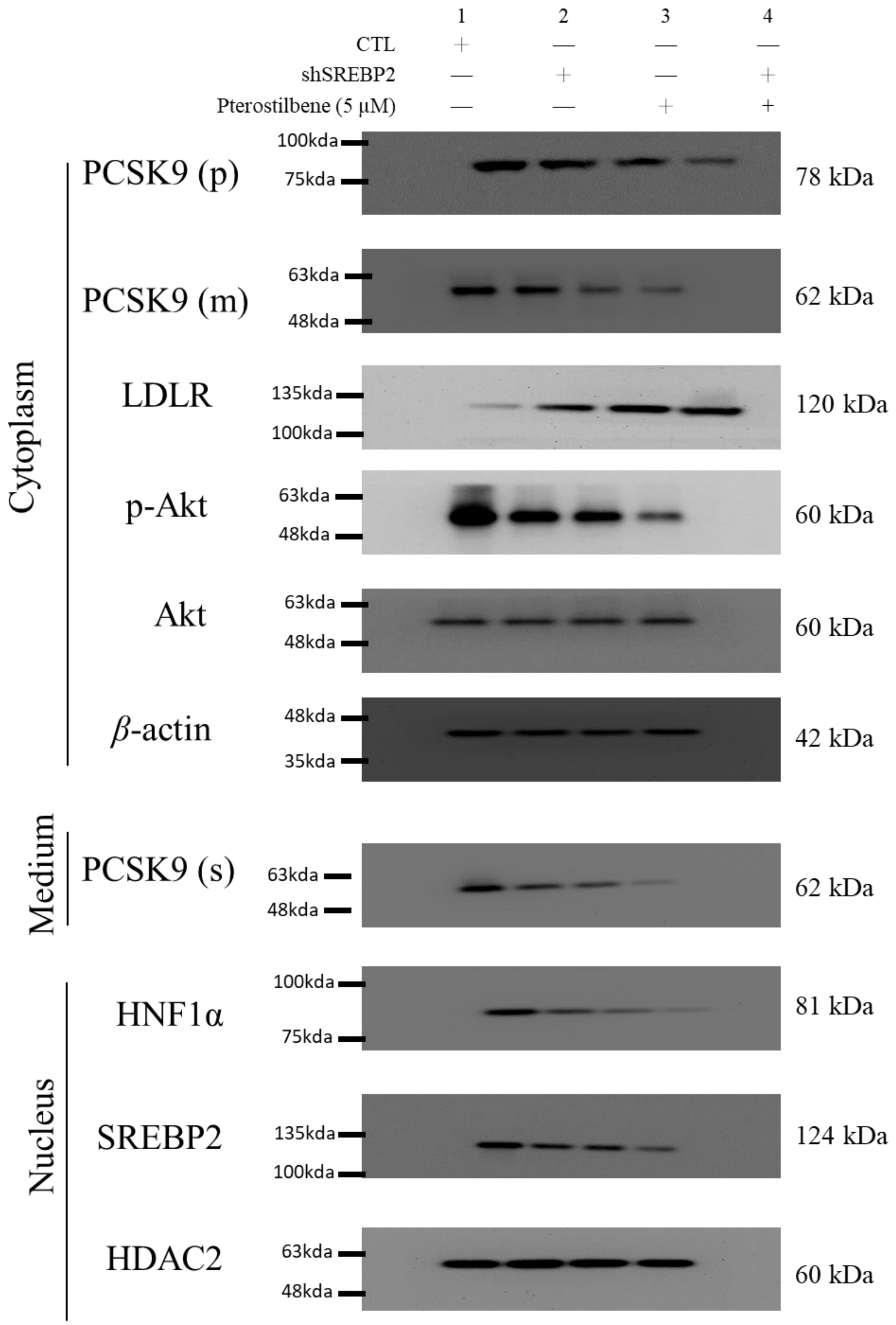

Supplementary Figure S4. Full-size blots of Figure 5A
